# A Stability-Indicating RP-HPLC Assay Method for 5-Fluorouracil

**DOI:** 10.4103/0250-474X.59544

**Published:** 2009

**Authors:** V. R. Sinha, R. V. Kumar, J. R. Bhinge

**Affiliations:** University Institute of Pharmaceutical Sciences, Panjab University, Chandigarh-160 014, India

**Keywords:** 5-Fluorouracil, analysis, excipients, formulation, HPLC, stability, stress studies, validation

## Abstract

The present study describes the development of a validated RP-HPLC method for the determination of 5-fluorouracil in presence of its degradation products or other pharmaceutical excipients. Stress studies were performed on 5-fluorouracil and it was found that it degrades sufficiently in alkaline conditions, while negligible degradation was observed in acidic, neutral, oxidative and photolytic conditions. The peaks of the degradation products were not observed in the chromatogram due to the nonchromophoric nature of the degradation moiety formed. The separations were carried out on a C-18 reversed phase column (Phenomenex; Prodigy ODS3V, 250×4.6 mm, 5 μ) using 50mM KH_2_PO_4_ (pH, 5.0) as mobile phase at a flow rate of 1.2 ml/min and temperature of 30°. The wavelength of detection was 254 nm. A retention time of nearly 6 minutes was obtained. Analytical validation parameters such as specificity and selectivity, linearity, accuracy and precision were evaluated. The calibration curve for 5-fluorouracil was linear (r^2^=0.999±0.0005) from range of 10 μg/ml to 100 μg/ml. Relative standard deviation values for all the key parameters, was less than 2.0 %. The recovery of the drug after standard addition to the degraded sample was found to be 104.69%. Thus, the developed RP-HPLC method was found to be suitable for the determination of 5-fluorouracil in bulk as well as stability samples of the pharmaceutical dosage forms containing various excipients.

Stability screening of drug candidates constitutes an inevitable part of drug discovery. The need for the stability studies on a drug candidate arises from the fact that the chemical integrity of the drug substance should be maintained until the compound is delivered to the intended site of action. Any form of chemical instability of the drug candidate may invariably affect the bioavailability and can further lead to toxic effects. Long term storage of the drug under various temperature and humidity conditions can affect its stability and this requires accurate methods to verify the apt storage conditions for the drug candidate[1].

International Conference on Harmonization (ICH) has made the need of a stability-indicating assay method (SIAM) for every drug candidate mandatory. A stability-indicating assay method helps in establishing the inherent stability of the drug which in turn provides assurance on detection changes in identity, purity and potency of the product on exposure to various conditions. In this study, the drug candidate is exposed to a variety of stress conditions like acidic, caustic, neutral, photolytic and oxidative stress[2,3]. As per the ICH guidelines stress testing of the drug substance aids in identifying the likely degradation products, which in turn can help in establishing the degradation pathways and the intrinsic stability of the molecule and validate the stability indicating power of the analytical procedures used.

5-Fluorouracil (5-FU), 5-fluoropyrimidine-2,4(1*H*,3*H*)-dione (fig. 1) is marketed mainly as intravenous solutions and has a very short metabolic half life of the order of 15 min[4]. It is one of the most widely used drugs for the treatment of metastatic carcinomas of the breast and the gastrointestinal tract. Determination of 5-FU and its metabolites in biological fluids using HPLC method has been reported[5]. A high-performance liquid chromatographic (HPLC) analysis of 5-FU and its major metabolite 5-fluoro-5,6-dihydrouracil after conventional and reduced test dose in cancer patients in plasma was reported by Bocci *et al*[6]. The determination of 5-FU using gas chromatographic-mass spectrometric (GC-MS) method in plasma has been also described[7,8]. A HPLC method has also been developed for the simultaneous determination of 5-FU and its prodrug, tegafur in dog plasma[9]. Though various analytical methods have been developed for analysis of 5-FU, none of them have been used for establishing the stability of the drug candidate under stress conditions, which would indicate the ability of the analytical method employed in determination of the drug in various pharmaceutical dosage forms.

**Fig. 1:**
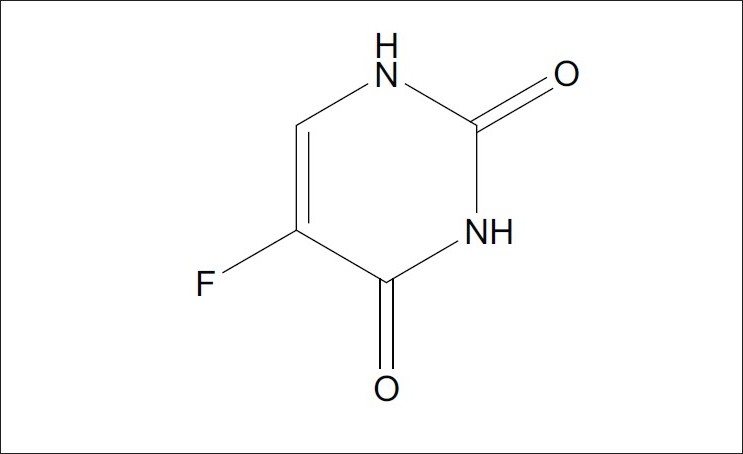
Structure of 5-FU

Thus, the present study was aimed for establishing a simple, accurate and rapid RP-HPLC method for the determination of 5-FU in presence of its degradation products or other pharmaceutical excipients. The method was validated following the analytical performance parameters suggested by ICH.

## MATERIALS AND METHODS

5-FU was received as a gift sample from Getwell Pharmaceuticals, Gurgaon, India. Sodium hydroxide and potassium dihydrogen phosphate were procured from S. D. Fine Chem. Ltd., Mumbai, India. Hydrochloric acid, dimethylformamide and hydrogen peroxide of analytical reagent grade were procured from Qualigens Fine Chemicals, Mumbai, India. Ultrapure water was prepared using ultrapure water purification set up supplied by Sartorius, Göttingen, Germany, which was further filtered through a 0.45 μ-filter.

### Chromatographic conditions:

The HPLC system (Shimadzu, Kyoto, Japan) consisted of LC-10AT pump, a SPD-10A UV/Vis detector and a DGU-14A degasser model. The separations were carried out on a C-18 reversed phase column (Phenomenex; Prodigy ODS3V, 250×4.6 mm, 5 μ). The column was operated at a temperature of 30°. Fifty millimoles KH_2_PO_4_ (pH 5.0) at a flow rate of 1.2 ml/min was used as the mobile phase. The wavelength of detection was 254 nm. The data were acquired and processed by CLASS-VP software (Shimadzu, Kyoto, Japan). Specificity studies were carried out using Waters Delta 600 HPLC equipped with a Waters 600 controller pump, Waters 2996 PDA detector and a degasser module from Waters, Milford, USA. Waters Empower 2 Software was used for data acquisition and processing.

### Stability studies:

Forced degradation studies under acidic, alkaline and neutral conditions were performed by refluxing using a heating mantle with temperature control (Tempad, Mumbai, India). The photostability studies were carried out in a stability chamber (KBF 240, WTB Binder, Tuttlingen, Germany) equipped with light sources as defined under option 2 of the ICH guideline Q1B. The light bank consisted of a combination of two black light Osram L73 lamps and four Osram L20 lamps. The blacklight lamp (L73) had a spectral distribution between 345 and 410 nm with maximum at 365 nm. The output of white fluoroscent lamps (L20) was similar to that specified in ISO 10977 (1993). Both UV and visible lamps were switched on simultaneously. The study was performed by keeping the samples at a distance of 9 inches from the light bank. The overall illumination at the point of placement was 5000 lux, which was tested using a calibrated lux meter (Escorp, New Delhi, India). The chamber was maintained at 40° and 75% RH.

### Validation:

Accurately weighed 100 mg of 5-FU powder was transferred to a 100 ml volumetric flask. To this 40 ml of water was added and then the mixture was sonicated for 30 s. The final volume was made up with water and the resulting solution was vortexed for 1 min.

### Hydrolytic studies:

Neutral hydrolysis of the drug was carried on solution prepared by dissolving 100 mg of the drug in water. The samples were exposed to stress conditions viz. 12 h, 1 d, 2 d, and 5 d reflux. Acid hydrolysis of the drug was carried out in presence of different concentrations of HCl (0.1N, 1N, 2N, and 5N)[1]. The solution was refluxed for 8 h (in 0.1N HCl), refluxed for 12 h (in 1N HCl), refluxed for 1 d (in 2N HCl) and refluxed for 2 d (in 5N HCl). While for alkaline hydrolysis 100 mg of the drug was dissolved in 100 ml of 0.1N NaOH and the resulting solution was refluxed for 8h. After exposure for the required duration of time the samples were diluted to a concentration of 100 μg/ml with the mobile phase (50 mM KH_2_ PO_4_). The samples were then injected into the HPLC system after filtration, through 0.45 μ-filters.

### Oxidative stress:

The drug solution was prepared by dissolving 100 mg of the drug in 100 ml of different concentrations of hydrogen peroxide solution (3, 10 and 27%). Initially the studies were carried out in 3% H_2_O_2_ for 6 h at room temperature and continued for 1 d at room temperature and finally the studies were also carried out in 10% and 27% H_2_O_2,_ for 1 d at room temperature[1]. After exposure for the required duration of time the samples were injected into the HPLC system after filtration.

### Photodegradation:

Some drug molecules undergo degradation upon exposure to light, which necessitate special storage conditions and protection from light. The official monograph directs that 5-FU should be protected from light, hence photodegradation studies were conducted that may provide information on the photostability of 5-FU under defined conditions, using different light sources. Sample solution of the drug was prepared by dissolving 100 mg of the drug in 100 ml of water to get a final concentration of 1 mg/ml. The samples were then kept in stability chamber (KBWF 240, WTB Binder, Germany) equipped with light source (option 2) as specified in the ICH guidelines Q1B and maintained at 40° and 75% RH. The samples of both solution and powder were kept in dark for the same period. Samples were withdrawn at different time periods and analyzed after sufficient dilution.

### Linearity:

Linearity of the method was established by preparing calibration curve in water. For this stock solution of drug (1 mg/ml) in water was prepared. Serial dilutions were then prepared ranging from 10 to 100 μg/ml. The solutions were then injected into the column and the corresponding area was obtained for each injection. From the area obtained, concentration and percentage relative standard deviation (% RSD) was calculated.

### Precision:

To determine the intra-day and inter-day precision of the method, repeatability studies were performed. The intra-day and inter-day precision studies were carried out on three different concentrations (20, 50 and 100 μg/ml). The samples were injected in hexaplicate on the same day and also on six different days. Concentration was calculated from the areas obtained and the results were expressed as %RSD.

### Accuracy:

Accuracy of the method was assessed by fortifying the degraded sample with known amount of the drug. Also five replicate injections of the working standard solution at a concentration of 100 μg/ml were made and % RSD was calculated.

### Specificity:

The specificity of the method was established through determination of the drug in the presence of degradation products/pharmaceutical excipients as well as the through determination of peak purity for the drug in the presence of degradation products/pharmaceutical excipients using PDA detector.

### Assay of pharmaceutical formulations:

The contents of ten indigenously developed delayed release enteric coated 5-FU tablets were thoroughly powdered and mixed, an amount of the powder equivalent to 250 mg of 5-FU was accurately weighed and added to 100 ml volumetric flask, 50 ml of dimethylformamide was added and the volume was made with the same. The resulting dispersion was sonicated for 15 min. The dispersion was kept as such for 12 h and vortexed for 5 min, dilution were made with the mobile phase, which were finally filtered through 0.22 μ-filter and analyzed. The same procedure was followed for the determination of 5-FU in stability samples (kept at 40°/75% RH for three months).

## RESULTS AND DISCUSSION

**Fig. 2:**
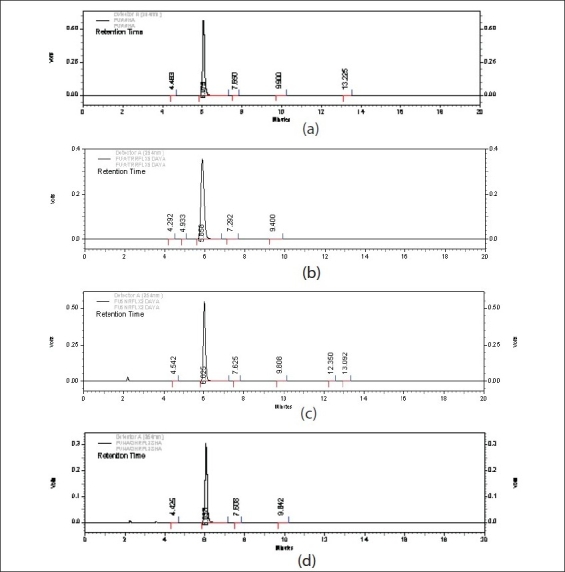
Chromatograms of 5-FU in hydrolytic stress conditions (a) Chromatogram of unstressed 5-FU samples on exposure to neutral hydrolysis (b) Chromatogram of stressed 5-FU samples on exposure to neutral hydrolysis (reflux, 5 days) (c) Chromatogram of stressed sample of 5-FU in 5 N HCl (refluxed for 2 d) (d) Chromatogram of stressed sample of 5-FU in 0.1 NaOH (refluxed for 8 h)

Degradation behavior of 5-FU under various stress conditions: The drug was found to be stable to neutral as well as acidic stress conditions. The amount left after 5-d reflux in water was found to be 94.89±0.72%, while in case of acid hydrolysis it was 101.88±0.82% (after 2 d reflux in 5N HCl, (fig. 2). The amount of the drug left after exposing to different neutral and acidic conditions is depicted in Table 1 and 2, respectively. In alkaline conditions the drug peak area decreased sufficiently. The results obtained on degradation of 5-FU in alkaline conditions indicated that around 45% of the drug degraded on refluxing it in 0.1N NaOH (pH~12.0) for 8 h. No new peaks were seen in the chromatograms of alkaline degraded samples of the drug (fig. 2) as compared to the standard. Some of the authors have reported that a nonchromophoric compound is formed on degradation of the drug which is not retained on the column and hence not detected[10,11]. Garrett *et al.* have reported the kinetics of hydrolysis of 5-halouracils in which they have said that at pH values greater than ten, these halouracils degrade with the possible formation of barbituric acid as a key intermediate which further degrades immediately into non-chromophoric products[12]. Thus, in case of 5-FU also degradation products formed on treatment with alkali were low molecular weight nonchromophoric compounds, which were not retained and hence no new peak was observed in the chromatogram.

**TABLE 1:** 5- FU EXPOSURE TO NEUTRAL HYDROLYSIS

Time of Exposure	Temperature	Amount Remaining (%)
12 h	Refluxing	101.92±0.27
1 d	Refluxing	98.28±2.70
2 d	Refluxing	100.03±0.61
5 d	Refluxing	94.89±0.72

Extent of decomposition observed in 5-FU (100 μg/ml) on exposure to neutral hydrolysis

**TABLE 2:** 5- FU EXPOSURE TO ACIDIC HYDROLYSIS

Strength of Acid (HCl)	Time of Exposure	Temperature	Amount Remaining (%)
0.1 N	8 h	Refluxing	99.79±2.57
1 N	12 h	Refluxing	96.76±2.11
2 N	1 d	Refluxing	99.35±0.88
5 N	2 d	Refluxing	101.88±0.82

Extent of decomposition observed in 5-FU (100 μg/ml) on exposure to acidic hydrolysis

**Fig. 3:**
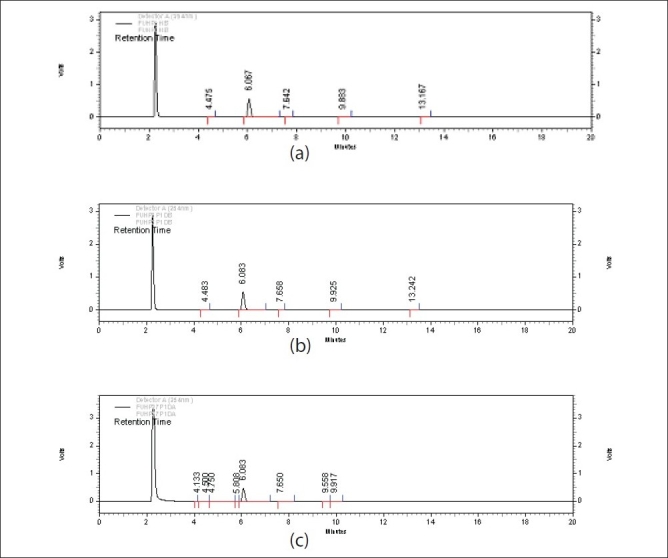
Chromatograms of 5-FU in oxidative stress conditions Chromatograms of stressed 5-Fluorouracil samples (a) Chromatogram of 5-FU (Rt: 6.067 min) initially after addition of 3% Hydrogen Peroxide (Rt: 2.3 min) (b) Chromatogram of 5-FU (Rt: 6.083 min) treated with hydrogen peroxide (3%) for 1 d at room temperature, (c) Chromatogram of 5-FU (Rt: 6.083 min) treated with hydrogen peroxide (27%) for 1 day at room temperature.

The drug was found to be stable to oxidative stress. However, nearly 15% drug degradation was observed on exposure to 27% H_2_O_2_ for 1 d (Table 3). No new peaks were seen in the chromatograms of degraded samples of 5-FU as compared to the standards (fig. 3). This indicated that the drug did not degrade to a sufficient extent and the degraded products formed were low molecular weight nonchromophoric compounds which were not retained and hence not detected. The results obtained indicate that the drug is quite stable under oxidative stress even after exposure to stringent conditions.

**TABLE 3:** 5-FU EXPOSURE TO OXIDATIVE STRESS AT ROOM TEMPERATURE

Strength of H_2_O_2_ (%)	Time of Exposure	Amount Remaining (%)
3	6 h	99.48±1.52
3	1 d	93.35±3.06
10	1 d	93.97±2.07
27	1 d	85.31±0.01

Extent of decomposition observed in 5-FU (100 μg/ml) on exposure to oxidative stress at room temperature

**Fig. 4:**
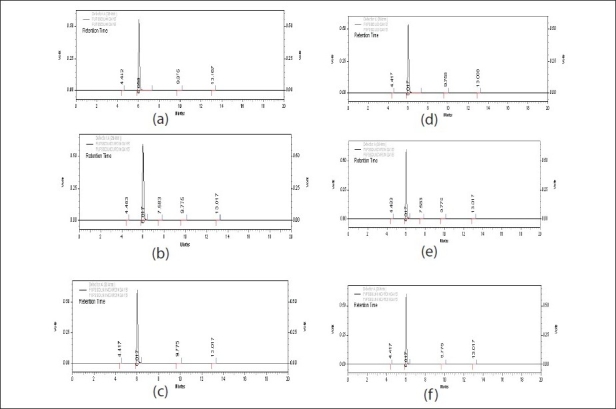
Chromatogram of the drug in different states exposed to photolytic stress. (a) 0 d (solid) (b) 10 d (solid, wrapped) (c) 10 d (solid, unwrapped) (d) 0 day (solution) (e) 10 d (solution, wrapped) (f) 10 d (solution, unwrapped)

No sufficient degradation of drug was observed even after 10 d of exposure both in solid and solution state (Table 4). No new peaks were seen in the chromatograms of the photodegraded samples of 5-FU as compared to the standards (fig. 4). Thus, the results obtained indicate that the drug is highly stable under photolytic stress even after stringent exposure conditions. The data obtained after linear regression analysis showed that the method was linear in the studied concentration range from 10-100 μg/ml. The values of slope and coefficient of correlation were found to be 41289±1.76 and 0.999±0.0005, respectively.

**TABLE 4:** 5-FU EXPOSURE TO PHOTOLYTIC STRESS BOTH IN SOLID AND SOLUTION STATE

State of drug	State of exposure	Time (d)	Amount Remaining (%)
		5	104.73±1.00
	Unwrapped		
		10	102.53±0.99
Solid		5	102.26±0.47
	Wrapped		
		10	101.12±0.02
		5	100.19±0.19
	Unwrapped		
		10	100.12±0.04
Solution		5	104.25±0.05
	Wrapped		
		10	98.98±0.01

Extent of decomposition observed in 5-FU (100 μg/ml) on exposure to photolytic stress both in solid and solution state (wrapped and unwrapped)

**Fig. 5:**
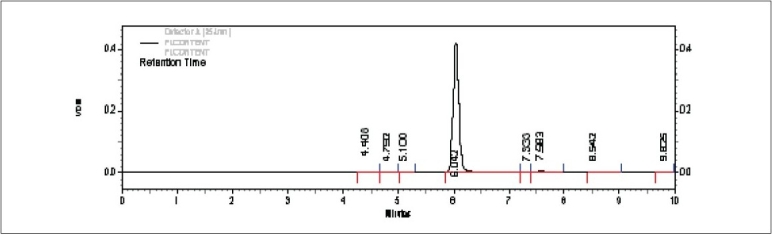
Chromatographic separation of 5-Flourouracil Separation of 5-Flourouracil from tablets subjected to accelerated conditions of 40°/75% RH for 3 months.

Peak purity studies on the drug peak using PDA detector showed purity angle (PA) value of 0.049 and purity threshold (TH) value of 0.259. As the purity angle value was found to be less than purity threshold the method was found to be specific to the drug. Studies performed on accelerated stability samples also showed good separation of drug peak from other peaks. This indicated that the method was specific to the drug (fig. 5).

The results of intra-day and inter-day precision studies are shown in Table 5. They revealed that % RSD values for intra-day studies ranged between 1.08 and 1.63% and for inter-day precision between 0.92 and 1.27%, which are within the permissible limits of 2.0%. The recovery of the drug after standard addition to the degraded sample (treated with 0.1N NaOH) was found to be 104.69%, which itself indicates good accuracy.

**TABLE 5:** PRECISION STUDIES

Actual concentration (μg/ml)	Measured concentration (μg/ml)±SD; RSD	

	Repeatability (n=6)	Intermediate precision (n=3)
20	20.51±0.24; 1.18	19.68±0.18; 0.92
50	52.15±0.86; 1.63	50.26±0.64; 1.27
100	100.13±1.08; 1.08	98.96±1.08; 1.07

SD stands for standard deviation and RSD denotes relative standard deviation

The amount of 5-FU in indigenously developed enteric-coated delayed release 5-FU tablet was found to be 100.01±1.58% (initially) and 99.10±0.62% (3 months, 40°/75%RH), with RSD of 1.58% and 0.63%, respectively. Stress testing of 5-FU under different conditions showed that the drug was stable to neutral and acidic hydrolysis but was susceptible to alkaline hydrolysis. Negligible degradation was observed in oxidative and photolytic conditions. In this study, intrinsic stability of 5-FU was established through employment of ICH recommended stress conditions. The drug was found to degrade sufficiently in alkaline conditions, while negligible degradation was observed under acidic and neutral hydrolytic, oxidative and photolytic stress conditions. The peaks of the degradation products were not observed in the chromatogram due to their nonchromophoric nature. The developed RP-HPLC method was found to be simple, rapid, sensitive, accurate, precise and specific, for the determination of 5-FU in bulk as well as stability samples of pharmaceutical dosage forms.
